# Predicting the impact of placing an overdose prevention site in Philadelphia: a mathematical modeling approach

**DOI:** 10.1186/s12954-021-00559-4

**Published:** 2021-10-30

**Authors:** Joanna R. Wares, Jing Dong, Jana L. Gevertz, Ami Radunskaya, Kendra Vine, Doug Wiebe, Sara Solomon

**Affiliations:** 1 Department of Mathematics and Computer Science, University of Richmond, 204 Jepson Hall, 221 Richmond Way, Richmond, VA 23173 USA; 2 Department of Mathematics and Statistics, The College of New Jersey, Ewing, NJ 08628 USA; 3 Department of Mathematics and Statistics, Pomona College, Claremont, CA 91711 USA; 4 Division of Substance Use Prevention and Harm Reduction, Department of Public Health, Philadelphia, PA 19109 USA; 5 Penn Injury Science Center, Department of Biostatistics, Informatics and Epidemiology, University of Pennsylvania, Philadelphia, PA 19104 USA

**Keywords:** Fatal overdose, Harm reduction, Markov model, People who use drugs, Supervised injection facility, Geospatial analysis, Overdose prevention site

## Abstract

**Background:**

Fatal overdoses from opioid use and substance disorders are increasing at an alarming rate. One proposed harm reduction strategy for reducing overdose fatalities is to place overdose prevention sites—commonly known as safe injection facilities—in proximity of locations with the highest rates of overdose. As urban centers in the USA are tackling legal hurdles and community skepticism around the introduction and location of these sites, it becomes increasingly important to assess the magnitude of the effect that these services might have on public health.

**Methods:**

We developed a mathematical model to describe the movement of people who used opioids to an overdose prevention site in order to understand the impact that the facility would have on overdoses, fatalities, and user education and treatment/recovery. The discrete-time, stochastic model is able to describe a range of user behaviors, including the effects from how far they need to travel to the site. We calibrated the model to overdose data from Philadelphia and ran simulations to describe the effect of placing a site in the Kensington neighborhood.

**Results:**

In Philadelphia, which has a non-uniform racial population distribution, choice of site placement can determine which demographic groups are most helped. In our simulations, placement of the site in the Kensington neighborhood resulted in White opioid users being more likely to benefit from the site’s services. Overdoses that occur onsite can be reversed. Our results predict that for every 30 stations in the overdose prevention site, 6 per year of these would have resulted in fatalities if they had occurred outside of the overdose prevention site. Additionally, we estimate that fatalities will decrease further when referrals from the OPS to treatment are considered.

**Conclusions:**

Mathematical modeling was used to predict the impact of placing an overdose prevention site in the Kensington neighborhood of Philadelphia. To fully understand the impact of site placement, both direct and indirect effects must be included in the analysis. Introducing more than one site and distributing sites equally across neighborhoods with different racial and demographic characteristics would have the broadest public health impact. Cities and locales can use mathematical modeling to help quantify the predicted impact of placing an overdose prevention site in a particular location.

## Background

The opioid epidemic in the USA is worsening, with overdose mortality rates continuing to rise [1]. Pennsylvania has the third highest overdose rate in the USA, with an annual rate of 36 per 100,000. Within PA, Philadelphia has an overdose death rate of 70 per 100,000, the highest among all counties in the state, and almost double the state average [2].

To address the opioid crisis, the city of Philadelphia created a task force and multi-pronged approach to address this public health problem. This included the Resilience Project [3], a collaboration of 35 city departments that worked together across seven key mission areas to address the opioid epidemic: (1) closing encampments and providing alternate housing and recovery services to its residents; (2) reducing criminal activity in areas with high drug activity; (3) reducing the number of unsheltered individuals; (4) reducing trash and litter including drug paraphernalia; (5) reducing overdoses and the spread of infectious diseases; (6) increasing treatment options; and (7) mobilizing community resources [3].

To date, the Resilience Project has had some successes: expanding emergency and temporary housing, hosting litter cleanups, distributing education about judicious opioid prescribing, increasing HIV testing, and increasing awareness about treatment availability, among other initiatives. However, despite progress, overdose rates remain high, with over 1100–1200 overdose deaths in Philadelphia annually since 2017. The crisis remains prominent with the COVID-19 pandemic introducing heightened challenges for people who use drugs [3, 4].

To address the wider opioid crisis, a collaborative group of city officials, local nonprofits, and community organizers came together to found Safehouse, a privately funded Pennsylvania nonprofit corporation whose mission is to save lives by providing a wide range of overdose prevention services throughout the city (https://www.safehousephilly.org/about). One innovative goal of Safehouse is to open multiple overdose prevention sites (OPSs) throughout the city. An OPS is a well-lit hygienic space where people can use illicit drugs under the supervision of trained health personnel. The main goal of OPSs is for trained health personnel to reverse injection drug overdoses when they occur. Secondarily, OPSs also educate clients about how to avoid injection-related harms, provide clean injecting supplies to reduce disease spread, and offer linkages to treatment and social services.

Although sites that exist in this fashion have operated successfully for over three decades in Europe, and for two decades in Australia and in Canada, the legality of OPSs in the USA has been a matter of intense debate [5, 6]. OPSs attract high-risk drug users and show positive outcomes in decreasing overdose fatalities, infectious disease spread, and public consumption, while also improving public amenity, increasing safe-injecting practices, promoting effective treatment methods, and yielding cost savings by preventing blood-borne infections and fatal overdoses. At the same time, research has shown that they do not increase drug use and related risks or neighborhood crime [7, 8]. Yet, attempts at opening such facilities in the USA have collided with federal drug laws. In 2019, a motion was filed to prevent Safehouse from opening OPSs in Philadelphia on the grounds that such facilities are illegal, and that they would promote rather than curb the use of opioids. In February of 2020, a federal judge entered a controversial final ruling that Safehouse's supervised injection site does not violate federal law. This ruling legally cleared the way for Safehouse to announce that the nation's first OPS would open in South Philadelphia. Other US cities, including Seattle, New York, San Francisco, and Somerville, Massachusetts, are also considering opening supervised injection sites [9].

The opening of OPSs poses several challenges, none more immediate than choosing the location to build such a facility. As soon as Safehouse was given legal clearance to build an OPS in South Philadelphia, backlash emerged from residents and politicians representing this neighborhood. As a result, Safehouse struggled to secure a site for their OPS, and on February 27, 2020, they announced that the opening of an OPS in Philadelphia would be delayed until the community could agree on the optimal location for a first site [9].

Given the controversy surrounding the opening of an OPS in Philadelphia, there is a clear need to present the community with compelling evidence of the efficacy and safety of such facilities. Just as importantly, the success of an OPS will be highly dependent on its location. If an OPS is built too far from people who use drugs, the facility is unlikely to be used and the benefits to society will be minimal. However, there are understandable desires to keep these facilities away from schools and other community locations, which imposes constraints on where such sites can be built.

The goal of this work is to use mathematical modeling to measure the potential impact of an OPS on overdose rates in the city of Philadelphia. Others have used dynamic models to study the opioid epidemic, both deterministically [10] and stochastically, including an optimal control study of a related stochastic compartment model [11, 12], a more detailed compartment model that aims to quantify the effect of nearly a dozen policy responses [13], a compartment model that investigated the impact of prescription drug monitoring programs [14], and an age-structured compartment model that focuses on the incidence of hepatitis C infections in young people who inject drugs [15]. Agent-based models of opioid use have also been developed [16, 17]. Unlike compartment models, these models are intended to capture more localized features of an epidemic. An excellent review of how computational approaches are more generally being applied to understand and improve opioid use disorder can be found in Griffin 2020 [18].

To our knowledge, simulation-based modeling has not been employed to explicitly measure the impact of an OPS. Herein, we formulate a Markov-type model of opioid use and analyze the model both with and without an OPS. Our model uses overdose location data to approximate where people who use opioids live and inject in the city of Philadelphia. Other parameters are chosen to correspond with data from other studies about OPS usage. After calibrating the model to existing data, we utilize the model to estimate the direct and indirect impact of placing an OPS in the Kensington neighborhood of Philadelphia. In particular, we investigate how many fewer fatal and nonfatal overdoses will occur under various assumptions and analyze the demographics of the people who will be affected.

## Methods

In this section, we describe in detail the mathematical model used to study the effect of establishing an OPS in an urban center. We parameterize our model using location-specific nonfatal (January 1 to December 31, 2018) and fatal (July 1, 2017 to June 30, 2018) opioid overdose data from the city of Philadelphia received from the Division of Substance Use Prevention and Harm Reduction of the Department of Public Health.

### Mathematical model

**Fig. 1:**
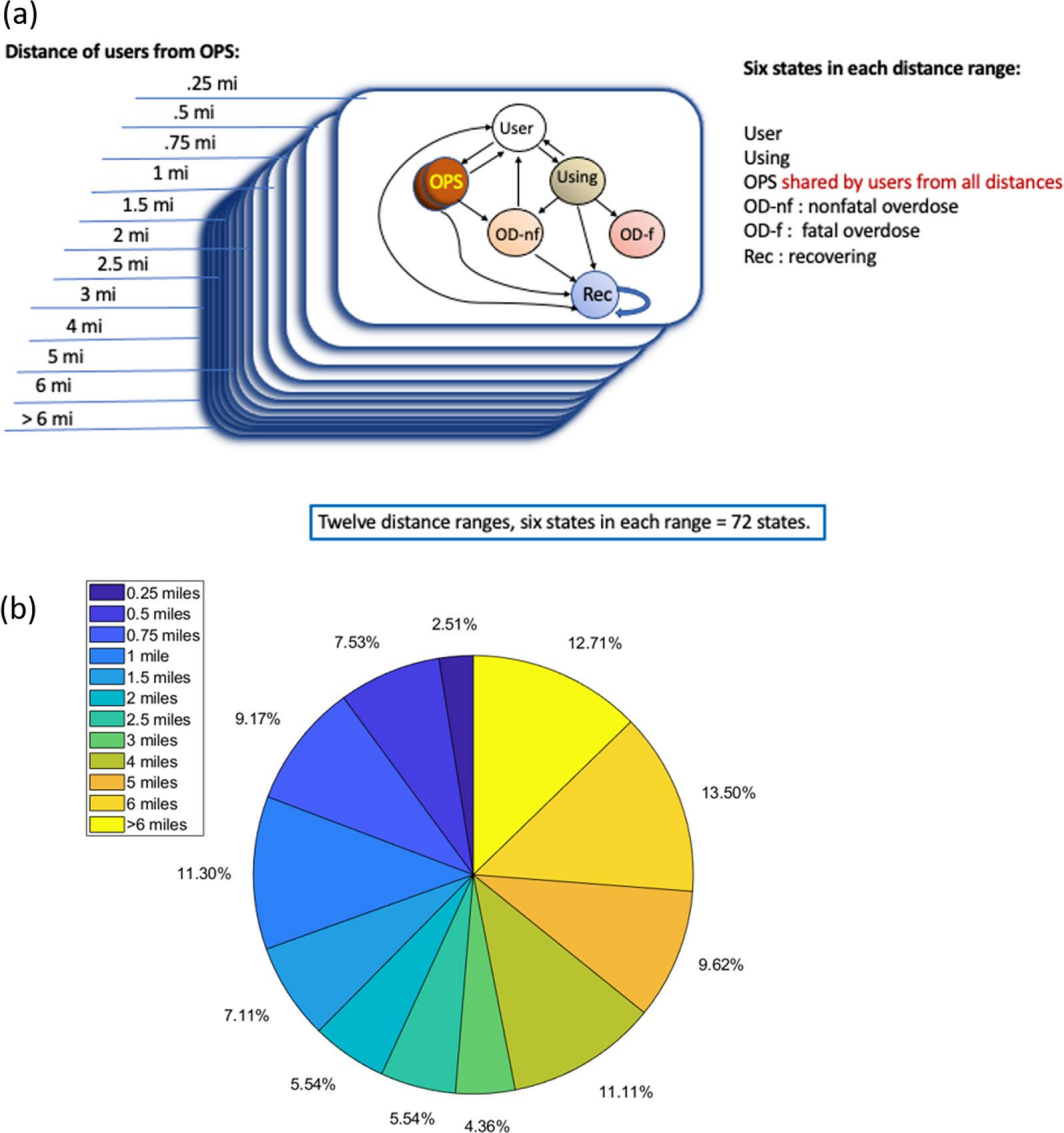
**a** Model schematic. **b** Percent of Philadelphia opioid users located within specified distance from proposed OPS site

The model is close to a discrete Markov chain, and so we call it a “Markov-type” model. It is a discrete-time, probabilistic mathematical model describing movement between a finite number of states. We divide opioid users into a discrete set of states. The model then assigns probabilities of people transitioning between states at each 30-min time step. A key characteristic of a Markov model is that these transition probabilities only depend on the current state, and this is true in our model, with one exception: the probability of transitioning into the “OPS” state is limited by the maximum capacity of the OPS. Our model assumes that people who use opioids can exist in one of six states (Fig. 1a):

- *State 1: Opioid users not currently using drugs* These individuals can decide to use drugs at the next time step, or to stay in the “not currently using” state. If they decide to use drugs, they may decide to try to do so at the OPS (State 3) or not (State 2). They may also decide to go from State 1 (this state) to a Treatment/Recovery state (State 4), where they are no longer using drugs unless they reenter State 1.
- *State 2: Using drugs outside of the OPS* From this state, an opioid user can overdose, either fatally (State 6) or non-fatally (State 5). If they do not overdose, they may go back to State 1, or they may go to the Treatment/Recovery state (State 4).
- *State 3: Using drugs in the OPS* Individuals who are in this state have the same transition options as those who are using drugs outside of the OPS (State 2). However, we assume that no fatal overdoses occur in the OPS [19]. Therefore, opioid users who are in the OPS can transition to the non-fatal overdose state (State 5), the Treatment/Recovery state (State 4), or back to State 1.
- *State 4: Treatment/Recovering (not currently using opioids)* Individuals in the Treatment/Recovery state can either stay in the Treatment/Recovery state at the next time step (stay at State 4) or start using opioids again and move back to the User state (State 1).
- *State 5: Non-fatal overdose state* Individuals in this state overdose, but do not die. We assume that all overdoses are non-fatal if they occur in the OPS. From this state, a user can either return to State 1, or go to Treatment/Recovery (State 4).
- *State 6: Fatal overdose state* Individuals in this state remain in this state. In the language of Markov Chains, State 6 is an *absorbing* state.

A schematic of these six states and the possible transitions between them is given in Fig. 1a. The Markov-type model is completely described by estimating the probabilities of transitioning between states (See Model Parameters).

We note a few additional modeling assumptions. First, we incorporate the observation that the probability of going to an OPS decreases with the user’s distance from the OPS [20]. Second, we account for the finite capacity of the OPS. If an OPS is full, a user is unable to enter. As a result, the probability of a transition to the OPS state (State 3) is a function of the number of individuals who use opioids who are trying to enter the site at that time step.

### Model parameters

Parameter values for the model were estimated from the overdose data described above and from the literature. Following news reports [21, 22], we conservatively estimated that there were approximately 55,000 regular opioid users in Philadelphia.

To estimate the percentage of opioid users that will enter treatment/recovery over a year, we compared sources. One source stated that “almost 21 million Americans have at least one addiction, yet only 10% of them receive treatment” [23]. This percent, however, is not specific to opioid use and is not a yearly statistic. A retrospective cohort study out of Massachusetts found that 34% of adults who survived an overdose from 2012 to 2014 took medication for their opioid use disorder within one year of their overdose [24]. This percentage likely overestimates the number of individuals using opioids who received medication, as it only tracks those who overdosed and were therefore forced to receive medical attention. Given these two data points, we estimated that 15% of opioid users seek treatment per year at baseline, but we also varied this parameter under other assumptions (see “Results”).

Although many individuals using opioids enter treatment/recovery each year, the NIH reports that approximately 40–60% of individuals with an addiction relapse. Opioid relapse rates tend to be higher than this [25], with some studies placing the relapse rate of opioid users as high as 80% [26]. For our purposes, we conservatively estimated that 50% of opioid users will relapse during each year.

Assuming that 55,000 opioid users enter treatment/recovery at a rate of 15% per year and relapse at a rate of 50% per year, the equilibrium number of opioid users in treatment/recovery at any time is nearly 13,000. The simulation was started with the equilibrium number of people in treatment/recovery. Under the guidance of Safehouse, we chose an expected location for the first OPS in the Kensington neighborhood of Philadelphia. The proportion of individuals using opioids and subjects in treatment/recovery in each location ring around the OPS was estimated from the proportions of overdoses in the respective locations (Fig. 1). We used non-intersecting distance rings around the OPS and set the percentage of opioid users in each ring proportional to the percentage of overdoses that occurred in these rings in the one-year period. The distances and percentages are summarized in Fig. 1b.

Following data from a safe injection facility in New York City, we assumed that the farther away a user is from the OPS, the less likely they were to try to enter the OPS to use drugs [20]. We fit an exponential distribution to the numerical data given in [20] to be able to predict the probability of trying to enter an OPS for other distances that were not measured in that work. The model further assumed that opioid users who were within 0.25 miles of the OPS had a 67% chance of attempting to go to the OPS to use drugs. Discretizing the exponential distribution to match the states in the model (the “distance rings” shown in Fig. 1), this probability was then reduced by a factor that depends on the distance from the OPS, resulting in the probabilities given in Table 1.

**Table 1:** Probability of visiting an OPS based on distance from site (data from Behrends et al., [20]), scaled by 0.67 to indicate that not all users within a fixed distance will attempt to enter an OPS

Distance from OPS (mi)	[0, .25]	(.25, .5]	(.5,.75]	(.75,1]	(1,1.5]	(1.5,2]
Probability of visiting	0.67	0.67 × 0.85	0.67 × 0.72	0.67 × 0.58	0.67 × 0.47	0.67 × 0.29
Distance from OPS (mi)	(2,2.5]	(2.5,3]	(3.5,4]	(4,5]	(5,6]	> 6
Probability of visiting	0.67 × 0.18	0.67 × 0.09	0.67 × 0.051	0.67 × 0.023	0.67 × 0.010	0.67 × 0.0027

We assumed that on average, users used drugs four times a day [27], and that overdose rates were estimated from the number of overdoses in the data and the assumption that there were 55,000 active opioid users. This led to rates of 6.9% of opioid users nonfatally overdosing per year and 1.88% of opioid users fatally overdosing per year. All rates were converted from yearly to half-hourly rates for the model. For the baseline model, we assumed that the maximum capacity of the OPS was 30, but this was varied in later experiments. These data came from the Division of Substance Use Prevention and Harm Reduction of the Department of Public Health and therefore likely represent an underestimate of the true rate that people overdose from opioid use. There are likely many more overdoses happening in the community that are not reported. Other studies suggest much higher overdose rates [28, 29], and it is also likely that the type of opioid used will influence this proportion [30]. Because of this underestimate of overdose rates, our results provide a lower bound for the effects of the overdose prevention site.

We assumed that the OPS would be open 20 h a day, 7 days a week. We also assumed that new users would enter the population at nearly the same rate as those that were fatally overdosing, here estimated at 3 per day on average.

## Results

### Summary of data

The age, gender, race, and ethnicity breakdown (where available) for the location-based Philadelphia overdose data are summarized in Table 2. There were 3788 reported nonfatal overdoses during the specified one-year period of time, with an average age of 41.5 years. Female opioid users were responsible for 27.6% of all overdoses. There was no breakdown by race or ethnicity for nonfatal overdoses.

**Table 2:** Demographics of nonfatal and fatal overdoses ((31) and data from the Division of Substance Use Prevention and Harm Reduction of the Department of Public Health)

	Gender	Race	Ethnicity
Nonfatal ODs	Male: 70.83% Female: 27.59%	N/A	N/A
Fatal ODs	Male: 73.48% Female: 26.52%	White: 69.70% Black: 29.33%	Hispanic: 12.78% Non-Hispanic: 86.16%
City Demographics	Male: 47.3 Female: 52.7%	White: 40.7% Black: 42.1%	Hispanic: 14.7% Non-Hispanic: 85.3%

There were 1033 reported fatal overdoses during the specified one-year period of time, with an average age of 43.91 years. Females and males made up 26.5% and 73.5% of these fatal overdoses, respectively. Nearly seventy percent (69.70%) of fatal overdoses are attributed to White individuals, though they represent only 41.2% of the city’s population ([31] and data from the Division of Substance Use Prevention and Harm Reduction of the Department of Public Health). In contrast, while Black individuals represent 42.3% of the City’s population, they composed only 29.3% of the fatal overdoses. Hispanic and Non-Hispanic individuals made up 12.8% and 86.2% of the fatal overdoses, respectively (Table 2).

**Fig. 2:**
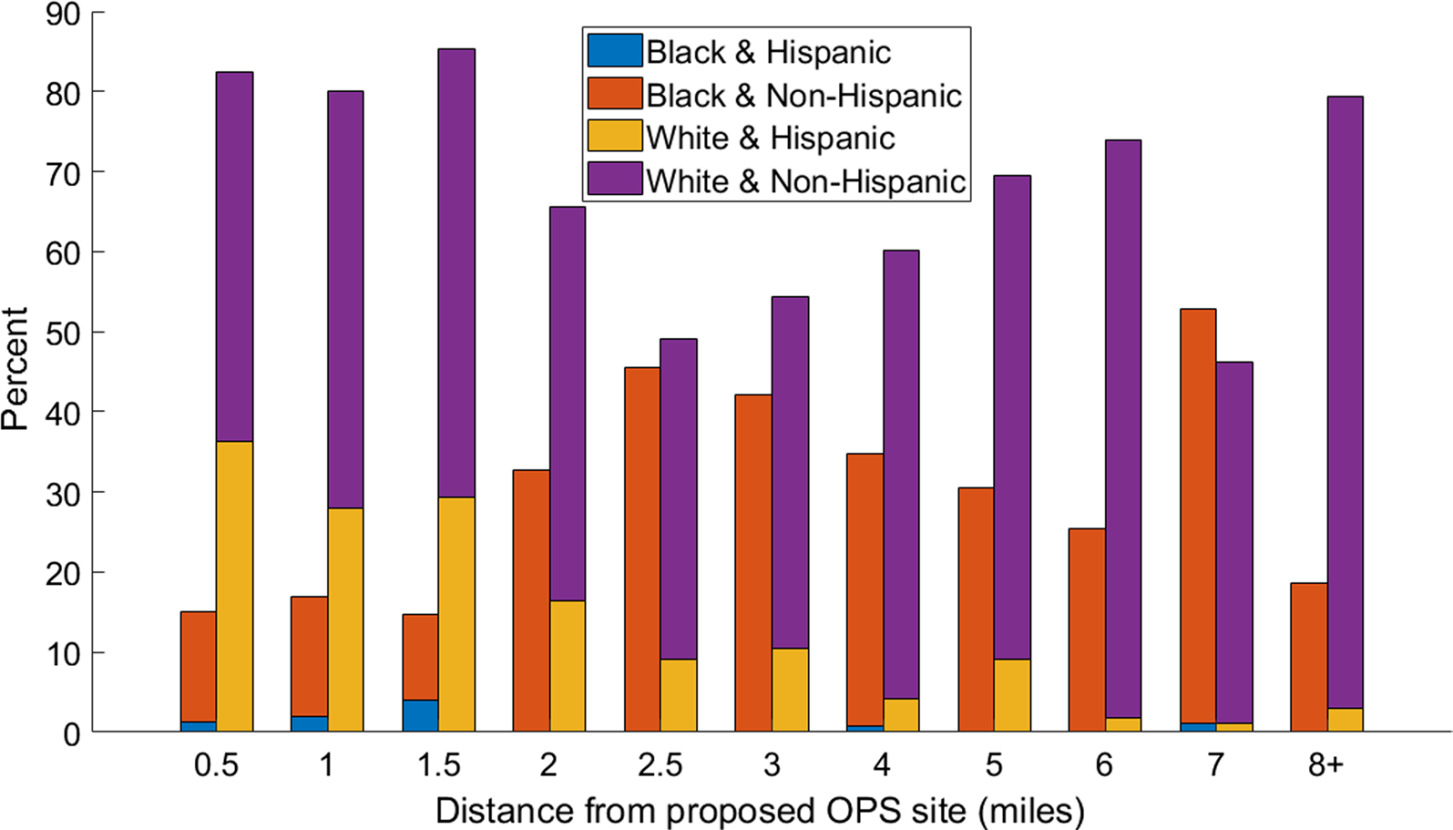
Demographics of individuals who overdosed based on distance from proposed OPS

Given that the use of an OPS is greatly influenced by proximity, we also looked at the identity and location of individuals who overdosed relative to the proposed OPS site. Figure 2 breaks down the demographics (race and ethnicity) of those who have overdosed based on their distance from the proposed OPS. What is evident is that the proposed site is more likely to benefit White opioid users as they represent over 80% of fatal overdoses that occurred within 1.5 miles of the proposed OPS (even though only 69.7% of fatal overdoses are in the White population). Similarly, the site also disparately benefits Hispanic opioid users as they represent over 30% of fatal overdoses that occur within 1.5 miles of the proposed site (even though only 12.8% of fatal overdoses are in the Hispanic population).

### Model calibration to data

All simulations were run for one year, with a time step of one-half hour. For a given set of parameters, 3000 replicate simulations were run, and the value reported is the end of year average value over these replicates.

Our first aim was to benchmark the model overdose results with the overdose data from Philadelphia. The data report 1033 individuals using opioids fatally overdosed in a one-year period and 3788 individuals using opioids nonfatally overdosed. Using the baseline parameter values described above with no OPS in place, our model predicts that 1045 opioid users will fatally overdose and 3933 will nonfatally overdose, numbers near the true values.

### Model prediction: direct effects of OPS

**Fig. 3:**
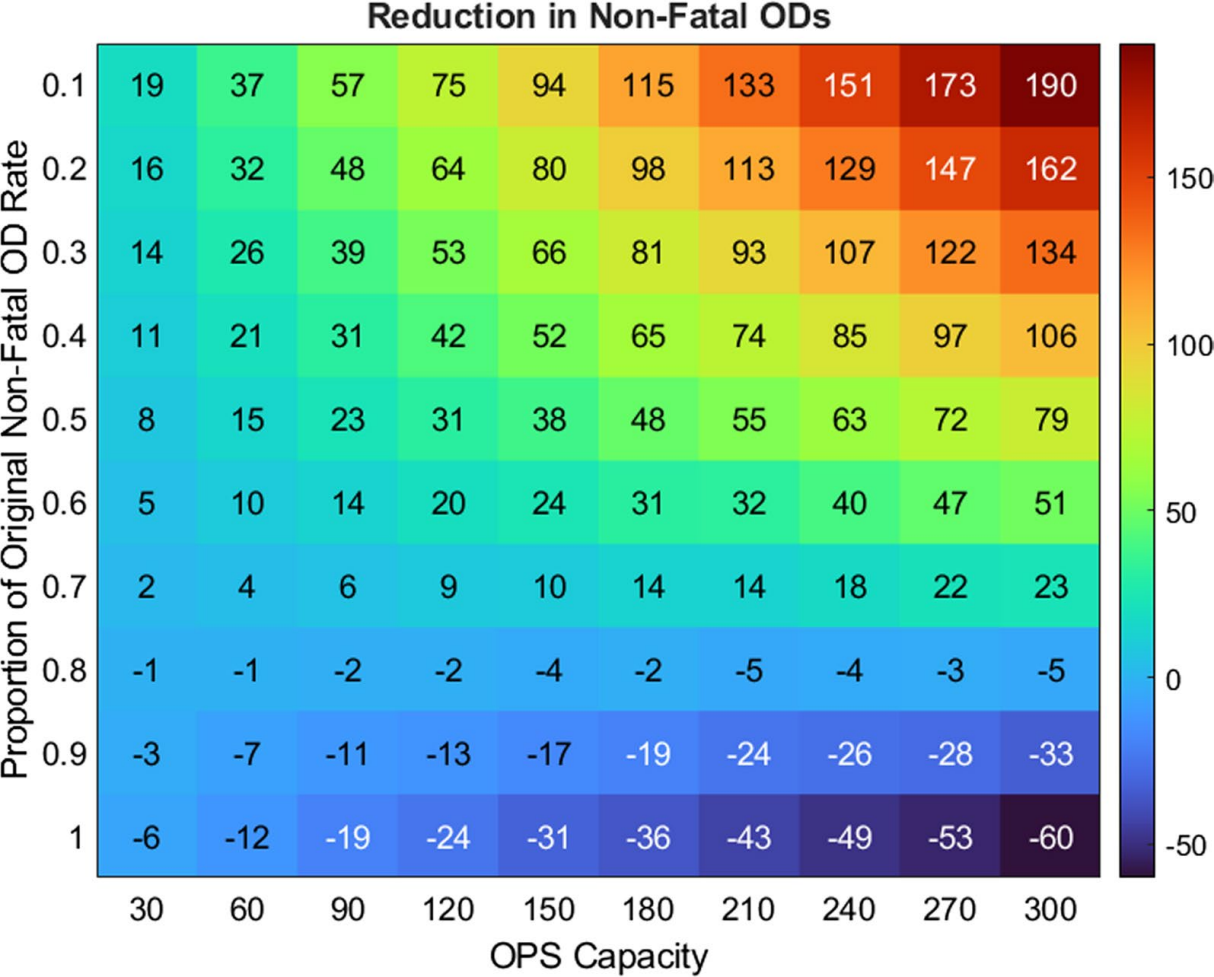
Top: Reduction in non-fatal overdoses as a function of OPS capacity and proportion of original non-fatal overdose rate in the OPS (in which individuals in OPS nonfatally OD at the same rate of those outside OPS). Positive numbers indicate a reduction in non-fatal ODs, whereas negative numbers indicate an increase (due to more opioid users surviving ODs)

OPSs around the world report no fatal overdoses occurring on their premises [32–34]. We therefore assume that any overdose that occurs inside of the OPS is a nonfatal overdose. In our first experiment, we assumed that the only effect of a 30-person capacity OPS was to eliminate fatal overdoses at the OPS. To model this assumption, we changed the nonfatal overdose rate in the OPS to be the sum of the fatal and nonfatal overdose rates in the population and set the fatal overdose rate in the OPS to zero. In this case, the model predicts that the OPS would reduce the fatal overdose rate by approximately 6 and 7 deaths per year while increasing the nonfatal overdose rate by about the same amount (Fig. 3, OPS = 30, proportion = 1). This increase in nonfatal overdoses occurs because the overall overdose rate does not change but instead, overdoses that occur in the OPS that would have been fatal are revived. An unexpected consequence of the decrease in fatal overdoses is that there are more active opioid users in the system over time. This allows for more total overdoses to occur, though in this particular scenario, the numbers are too small to make a noticeable difference.

In our next experiment, we varied the maximum capacity of the OPS from 30 to 300, in increments of 30 (Fig. 3, proportion = 1). We see an approximately linear reduction of 6 fatalities per year for every 30 extra seats in the OPS. Results suggest that increasing the capacity of the facility to treat 300 opioid users simultaneously would result in reducing fatal overdoses by over 58 while increasing nonfatal overdoses by a similar (but slightly larger) amount, 60. The larger increase in nonfatal overdoses versus reduction in fatal overdoses is likely due to having more individuals using opioids over time since less people are dying.

Since opioid users near the OPS are more likely to use drugs there instead of on the street or at home, the placement of the OPS will have a larger effect on the population living in close proximity to the site. Determining where to place the OPS will also determine who is being helped most. Out of the 58 fatalities that were averted when the max capacity was 300, 43 out of 58 (74%) revived in the OPS lived within a 1-mile radius of the OPS, though only 30% of all individuals using opioids lived within a 1-mile radius. Further, 50 of the 58 (86%) that were revived in the OPS lived within a 1.5-mile radius of the OPS while individuals in this region only made up 38% of all of the opioid users in Philadelphia.

In our simulations, the OPS is always full because of the large number of opioid users compared with available stations in the OPS. We expect that increasing the availability of OPS resources would reduce fatalities from opioid use linearly until resource availability better matches demand.

### Model prediction: indirect effects of the OPS on overdose

OPSs provide more services than just reviving individuals who have overdosed. They also provide safer spaces to inject, can test drugs for more deadly unwanted additions like fentanyl, and provide safety instructions and clean instruments to opioid users before they use opioids. These services are likely to reduce the overall overdose rate in the OPS, including the nonfatal overdose rate. To determine the effects of these safety precautions on the overall number of nonfatal overdoses seen in a year, we varied the nonfatal overdose rate in the OPS (Fig. 3, changing proportion). As an example, setting the proportion equal to 0.6 means the non-fatal overdose rate is 60% the rate without an OPS. This is in comparison with the prior section where only direct effects were considered, and therefore, the non-fatal overdose rate was fixed at the value seen without an OPS. In combination with this, we also varied the maximum capacity of the OPS to determine how the total number of nonfatal overdoses is affected by the ability to serve more clients (Fig. 3). Fatal overdoses are not changed by varying the rate of overdoses in the OPS because we assumed that all individuals who overdose can be revived in the OPS. Recall that in the model, the baseline overdose rate for the OPS is the nonfatal plus the fatal overdose rate since we assumed that all fatal overdoses become nonfatal in the OPS. Also recall that this led to an increase in nonfatal overdoses over time if the OPS is placed in the city.

In our previous simulation, the only effect of the OPS was to change fatal overdoses to nonfatal overdoses by reviving those individuals who overdose in the OPS. Because of this, when fatal overdose rates were reduced, we saw a similar *increase* in the nonfatal overdose rate. In the next set of simulations, we assumed that using opioids in the OPS is safer than outside, and we incrementally reduce the nonfatal overdose rate in the OPS. We found that if the interventions of the OPS reduce the overall overdose rate in the OPS by 30% (Fig. 3, proportion = 0.7), the overall effect of the OPS will be a reduction in the total number of nonfatal overdoses in the whole population. Since increasing the maximum capacity alone increases the number of nonfatal overdoses, and since reducing the overall overdose rate in the OPS decreases the number of nonfatal overdoses, we wondered if the two effects would balance at some point. Varying the two parameters together showed the point where the effects balanced out, here between 20 and 30% reduction in nonfatal overdose rate in the OPS, for any OPS capacity between 30 and 300.

### Model prediction: increasing treatment/recovery rate for all individuals who use opioids

Another aspect of an OPS is the education provided to clients about entering treatment/ recovery [35]. The OPS will serve as a safe place for opioid users, as well as a place where they can find resources for entering treatment/recovery and for general safer use practices. As a first pass at estimating the overall effect on the community from having an OPS, we varied the overall rates of treatment/recovery for the entire population. Here, we assume that by having an OPS in the city, the overall rates of treatment/recovery will increase. In the baseline simulation, we assumed that 15% of opioid users would go to treatment/recovery over a one-year period and that 50% of those would leave treatment/recovery and start using again (see Model Parameters). Assuming that the overall rate at which individuals using opioids move to treatment/recovery increases to 20% (from 15%) per year, the model predicts that there would be 90 fewer nonfatal overdoses and 23 fewer fatal overdoses in total (see Fig. 3b). If instead, the OPS could increase the overall treatment/recovery rate through education to 25% per year (from 15% per year), there would be 181 fewer nonfatal overdoses and 47 fewer fatal overdoses. Here, we kept the assumption that 50% of these individuals would leave treatment over the year and start using opioids again. In general, increasing the general population’s treatment/recovery rate by having an OPS in the city has a profound effect on the rates of overdose. The more the OPS can do to educate the general public, the larger the effect will be.

## Discussion

In this study, we predicted the effects of placing an overdose prevention site (OPS) in the Kensington neighborhood of Philadelphia, PA. We utilized data from the Philadelphia Department of Public Health describing overdoses over a one-year period. Under the assumption that there are 55,000 active opioid users, we found from the overdose data that approximately 6.9% of opioid users nonfatally overdose and 1.88% fatally overdose in a one-year period in the absence of an OPS in Philadelphia.

We then used these statistics, in combination with overdose location data, to build a Markov-type model to investigate the dynamics of drug use, overdose, and treatment/recovery assuming an OPS is present in the Kensington neighborhood of Philadelphia. We found that the direct effect of having an OPS, limited to only those that would be revived in the OPS, was small if the capacity of the OPS was limited to 30 individuals, but increased linearly with increasing capacity.

**Fig. 4:**
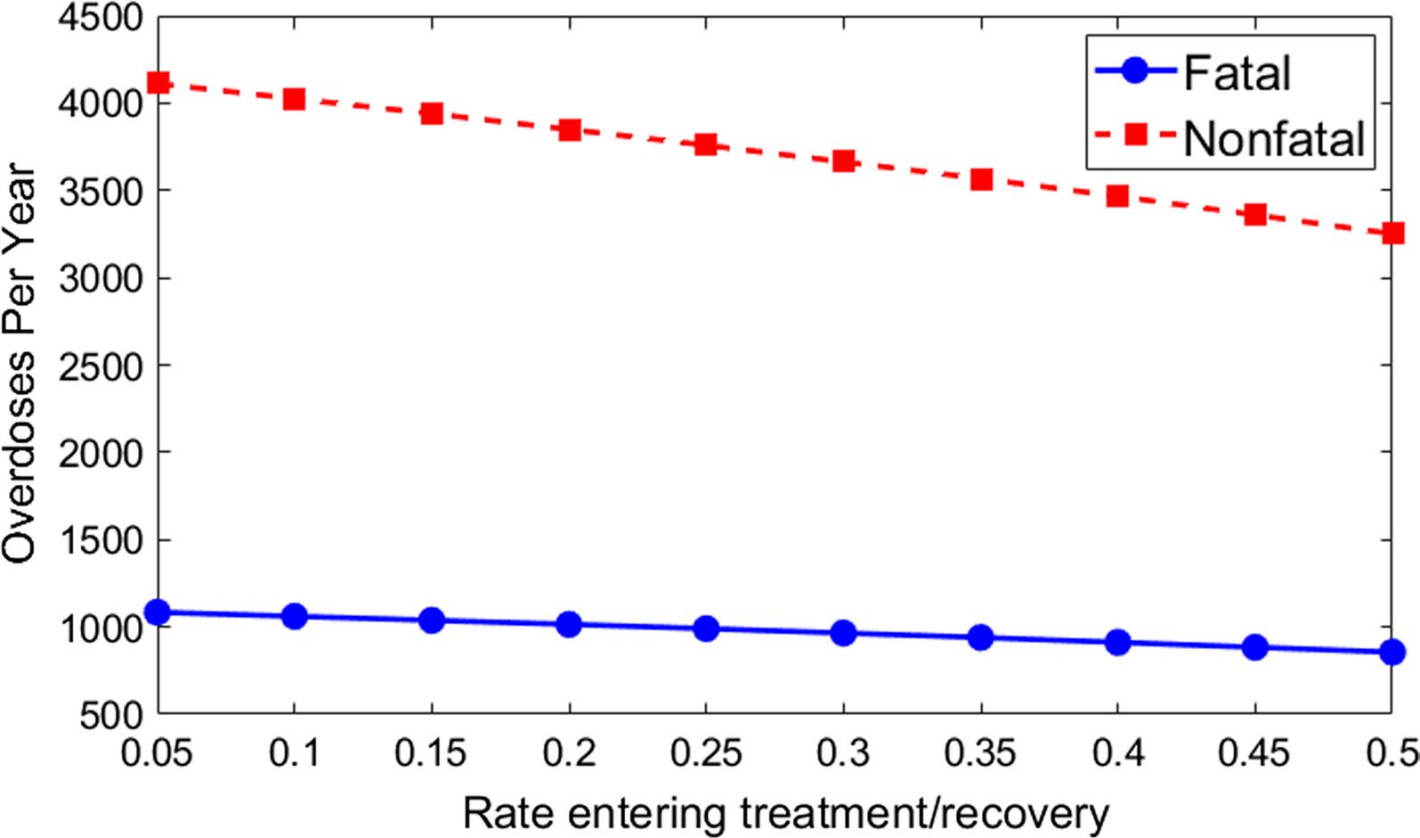
Number of fatal (blue) and nonfatal (red) overdoses per year as a function of rate of entering treatment or recovery for total population

If we included certain indirect effects of the OPS such as providing a safer environment, fentanyl testing, and education about safer use and treatment/recovery in our analysis, we found that the inclusion of an OPS could result in a much greater reduction in fatal overdoses. As shown in Fig. 3, nonfatal overdoses in the total population could be reduced if, for example through the testing of drugs, supervision, and other interventions, the OPS provided just a 20–30% reduction in overdoses, relative to the rate of overdoses outside the OPS. Our results suggest that the biggest benefit of the OPS would come from indirect effects that would assist the entire user population of Philadelphia. Exploring increased treatment/recovery rates of the total population (Fig. 4), we found that overdose rates were markedly reduced when user entrance into treatment/recovery increased from 15 to 20 percent per year. OPS education would likely cause other positive indirect effects such as disease reduction. In addition, the OPS could have other indirect effects such as lower contamination rates and lower rates of HIV transmission. In future work, we plan to explore these in an agent-based model where we can track which opioid users have been to the OPS and when.

Location of OPS placement will affect which individuals are helped the most, since those nearest to the OPS will be most likely to utilize the facility. Importantly, all analysis in this paper is based on the placement of the OPS in the Kensington neighborhood of Philadelphia with a maximum capacity of 300. If the OPS is placed at the proposed location in Kensington, the overdose data suggests that the OPS will disproportionality benefit White opioid users, as they represent over 80% of the fatal overdoses that occurred within 1.5 miles of the proposed site (as compared to being 69.7% of all individuals who use opioids in the city). Our simulations suggested that of those revived in the OPS, 86% of them would come from an area within 1.5 miles radius of the proposed OPS. We also plan to use the agent-based model to solidify our understanding of which individuals will be most helped by placing the OPS in various locations, as well as the demographic effects and cumulative impact of multiple OPSs in various locations in the city.

## Conclusions

The opioid epidemic continues to be an escalating problem in the USA. The COVID-19 pandemic introduced heightened challenges [4] for people who use drugs and paused some of the traction around the opening of an OPS. In addition, barriers to legally opening an OPS in the USA remain [36]. As cities such as Philadelphia begin to reopen, there may be an increased effort to open an OPS as part of a multifaceted response to rising rates of fatal overdose. This research lends insight not only to the direct and indirect benefit of an OPS but offers specific implementation considerations to optimize impact. Specifically, cities such as Philadelphia that are considering an OPS would benefit from multiple sites with sizable capacities. This would not only reduce the direct rate of fatal overdose, but also disperse services equally across neighborhoods with varying racial and ethnic characteristics. In addition, consistent with the model proposed by Safehouse, offering educational services and additional harm reduction strategies, such as fentanyl and syringe exchange, in conjunction with a safe place to use drugs reaches people who use drugs both indirectly and directly, thereby providing the greatest public health impact.

## Data Availability

Data used in this study include fatal and nonfatal incidents of overdoses over a one-year period (2018) from the City of Philadelphia, Department of Public Health. Data were retrieved using a data request form submitted to the Department of Public Health: https://www.phila.gov/services/mental-physical-health/medical-professionals/request-health-data/.

## References

[1] Staff. CDC: Morbidity and Mortality Weekly Report [Internet]. Centers for Disease Control and Prevention. Available from: https://www.cdc.gov/mmwr/volumes/67/wr/mm675152e1.htm?s_cid=mm675152e1_w

[2] Staff. CDC: 2019 Drug Overdose Death Rates. Centers Dis Control Prev [Internet]. Available from: https://www.cdc.gov/drugoverdose/deaths/2019.html

[3] Philadelphia Resilience Project. Philadelphia Resilience Project: Recovering Together [Internet]. 2019. Available from: https://www.phila.gov/media/20190619122049/Resilience-Report-06-2019.pdf

[4] Khatri UG, Perrone J (2020). Opioid use disorder and COVID-19: crashing of the crises. J Addict Med.

[5] Lofaro RJ, Miller HT (2021). Narrative politics in policy discourse: the debate over safe injection sites in Philadelphia, Pennsylvania. Contemp Drug Probl.

[6] Corbett A (2021). The locality’s case for safe injection facilities: legal obstacles and ways to overcome them. Univ Pennsylvania J Law Soc Chang..

[7] Sharfstein JM, Olsen Y. How not to spend an opioid settlement. 323. 2020 10.1001/jama.2020.1371 32181838

[8] Levengood TW, Yoon GH, Davoust MJ, Ogden SN, Marshall BDL, Cahill SR, et al. Supervised Injection Facilities as Harm Reduction: A Systematic Review. American Journal of Preventive Medicine. 2021. 10.1016/j.amepre.2021.04.017 PMC8541900 34218964

[9] Staff NBC. Judge Paves Way for Nation’s 1st Supervised Injection Site in South Philly . NBC Philadelphia [Internet]. Available from: https://www.nbcphiladelphia.com/news/local/judge-says-philly-safe-injection-site-would-not-violate-law/2306910/

[10] Battista NA, Pearcy LB, Strickland WC. Modeling the prescription opioid epidemic. Bull Math Biol. 2019;81(7). 10.1007/s11538-019-00605-0 31012032

[11] Befekadu GK (2019). On the asymptotic exit control problem for stochastically perturbed prescription opioid epidemic models. IFAC-PapersOnLine..

[12] Befekadu GK, Zhu Q (2019). Optimal control of diffusion processes pertaining to an opioid epidemic dynamical model with random perturbations. J Math Biol.

[13] Pitt AL, Humphreys K, Brandeau ML (2018). Modeling health benefits and harms of public policy responses to the US opioid epidemic. Am J Public Heal.

[14] Chen Q, Larochelle MR, Weaver DT, Lietz AP, Mueller PP (2019). Prevention of prescription opioid misuse and projected overdose deaths in the United States. JAMA Netw Open.

[15] Gicquelais RE, Foxman B, Coyle J, Eisenberg MC (2019). Hepatitis C transmission in young people who inject drugs: insights using a dynamic model informed by State Public Health Surveillance. Epidemics.

[16] Bobashev G, Goree S, Frank J, Zule W. Pain Town, an Agent-Based Model of Opioid Use Trajectories in a Small Community. In: Thomson R, Dancy C, Hyder Ayazand Bisgin H, editors. Social, Cultural, and Behavioral Modeling. Springer International Publishing; 2018. p. 274–85.

[17] Nelson AE. Quantifying spatial potential access equity in an agent based simulation model of buprenorphine treatment policy in the United States. [Portland, OR]: Portland State Univ.; 2018.

[18] Griffin PM (2020). Engineering approaches for addressing opioid use disorder in the community. Annu Rev Biomed Eng.

[19] Kerr T, Tyndall MW, Lai C, Montaner JSG, Wood E (2006). Drug-related overdoses within a medically supervised safer injection facility. Int J Drug Policy.

[20] Behrends CN, Paone D, Nolan ML, Tuazon E, Murphy SM, Kapadia SN (2019). Estimated impact of supervised injection facilities on overdose fatalities and healthcare costs in New York City. J Subst Abuse Treat.

[21] Lattanzio V. Philadelphia Launches Task Force to Combat Opioid and Heroin Crisis, Sets 90-Day Goal to Make Recommendations [Internet]. NBC Philadelphia. 2017. Available from: https://www.nbcphiladelphia.com/news/local/philadelphia-launches-opioid-and-heroin-task-force-sets-90-day-goal-to-make-recommendations/11759/

[22] Percy J. Trapped by the ‘Walmart of Heroin.’ New York Times. 2018;

[23] Staff. Addiction Statistics [Internet]. Addiction Center. Available from: https://www.addictioncenter.com/addiction/addiction-statistics/

[24] Larochelle MR (2018). Medication for opioid use disorder after nonfatal opioid overdose and association with mortality: a cohort study. Ann Intern Med.

[25] Staff. Opiate Addiction Recovery Statistics [Internet]. Alta Mira Recovery Programs. Available from: https://www.altamirarecovery.com/opiate-addiction-recovery-statistics/

[26] Clark PA, Lee MJ, Gulati S, Minupuri A, Patel P, Zheng S (2018). Comprehensive user engagement sites (CUES) in Philadelphia: a constructive proposal. Internet J Public Heal.

[27] Darke S, Mattick RP, Degenhardt L, Neale J (2003). The ratio of non-fatal to fatal heroin overdose [2] (multiple letters). Addiction.

[28] Maloney E, Degenhardt L, Darke S, Nelson EC. Are non-fatal opioid overdoses misclassified suicide attempts? Comparing the associated correlates. Addict Behav. 2009;34(9). 10.1016/j.addbeh.2009.04.011 PMC2730501 19447563

[29] Darke S, Ross J, Hall W (1996). Overdose among heroin users in Sydney, Australia: I. Prevalence and correlates of non-fatal overdose. Addiction.

[30] Roxburgh A, Darke S, Salmon AM, Dobbins T, Jauncey M (2017). Frequency and severity of non-fatal opioid overdoses among clients attending the Sydney Medically Supervised Injecting Centre. Drug Alcohol Depend.

[31] Staff. US Census: QuickFacts Philadelphia city, Pennsylvania [Internet]. United States Census. Available from: https://www.census.gov/quickfacts/philadelphiacitypennsylvania

[32] Ng J, Sutherland C, Kolber MR. Does evidence support supervised injection sites? Canadian Family Physician. 2017;63 PMC5685449 29138158

[33] Kral AH, Lambdin BH, Wenger LD, Davidson PJ (2020). Evaluation of an unsanctioned safe consumption site in the United States. N Engl J Med.

[34] Milloy MJS, Kerr T, Tyndall M, Montaner J, Wood E (2008). Estimated drug overdose deaths averted by North America’s first medically-supervised safer injection facility. PLoS ONE.

[35] Potier C, Laprévote V, Dubois-Arber F, Cottencin O, Rolland B (2014). Supervised injection services: What has been demonstrated? A systematic literature review. Drug Alcohol Depend.

[36] Tanenbaum M. Philly plan for opioid overdose prevention site shot down in federal court reversal [Internet]. Philly Voice. 2021. Available from: Philly plan for opioid overdose prevention site shot down in federal court reversal

