# Correction to: Predicting the impact of placing an overdose prevention site in Philadelphia: a mathematical modeling approach

**DOI:** 10.1186/s12954-021-00577-2

**Published:** 2021-12-15

**Authors:** Joanna R. Wares, Jing Dong, Jana L. Gevertz, Ami Radunskaya, Kendra Viner, Doug Wiebe, Sara Solomon

**Affiliations:** 1 Department of Mathematics and Computer Science, University of Richmond, 204 Jepson Hall, 221 Richmond Way, Richmond, VA 23173 USA; 2 Department of Mathematics and Statistics, The College of New Jersey, Ewing, NJ 08628 USA; 3 Department of Mathematics and Statistics, Pomona College, Claremont, CA 91711 USA; 4 Division of Substance Use Prevention and Harm Reduction, Department of Public Health, Philadelphia, PA 19109 USA; 5 Penn Injury Science Center, Department of Biostatistics, Informatics and Epidemiology, University of Pennsylvania, Philadelphia, PA 19104 USA

## Correction to: Harm Reduct J (2021) 18:110 https://doi.org/10.1186/s12954-021-00559-4

Following publication of the original article [1], the author name “Kendra Viner” was incorrectly written as “Kendra Vine.” This has been corrected with this erratum.

The original article has been corrected.
